# Amyand’s hernia in an 11-month-old: a rare intraoperative finding of an adherent appendix

**DOI:** 10.1093/jscr/rjag218

**Published:** 2026-04-04

**Authors:** Anish Sah, Bishal Budha, Shailesh Kurmi, Roshan Chaudhary, Jyoti Kalwary, Sanjeev Jha, Roshan Kapari, Manish Kumar Sah

**Affiliations:** Maharajgunj Medical Campus, Institute of Medicine, Tribhuvan University, Maharajgunj, Nepal; Maharajgunj Medical Campus, Institute of Medicine, Tribhuvan University, Maharajgunj, Nepal; Maharajgunj Medical Campus, Institute of Medicine, Tribhuvan University, Maharajgunj, Nepal; Maharajgunj Medical Campus, Institute of Medicine, Tribhuvan University, Maharajgunj, Nepal; Kathmandu Medical college and Teaching Hospital, Kathmandu, Nepal; Maharajgunj Medical Campus, Institute of Medicine, Tribhuvan University, Maharajgunj, Nepal; Maharajgunj Medical Campus, Institute of Medicine, Tribhuvan University, Maharajgunj, Nepal; Manipal College Of medical Sciences, Pokhara, Nepal

**Keywords:** Amyand’s hernia, inguinal hernia, appendectomy, infant

## Abstract

Amyand’s hernia is rare type of inguinal hernia characterized by the presence of the appendix within the hernia sac. It is more common in children due to patent processus vaginalis. It is often diagnosed intraoperatively, which makes it clinically significant, as it can mimic other groin pathologies and influence surgical decision-making. We report the case of an 11-month-old male who presented with a reducible right inguinal swelling since birth. On physical examination, a soft, non-tender mass was palpable in the right inguinal region, which increased in size with Valsalva-like maneuvers. Radiological findings confirmed the right inguinal hernia, but appendix was not visualized. During elective herniotomy, the sac was found to contain the cecum with a densely adherent, normal appendix. An appendectomy was performed along with herniotomy.

## Introduction

An inguinal hernia is a type of hernia in which there is protrusion of abdominal cavity contents through the inguinal canal. The inguinal hernia sac contains only the omentum and small intestine; however, in some unusual conditions, it may also contain ovary, fallopian tube, colon, and urinary bladder [1]. Amyand’s hernia is a very rare form of inguinal hernia in which there is presence of vermiform appendix in the hernia sac^2^. The incidence of Amyand’s hernia varies from 0.5% to 1% [2]. Due to patent processus vaginalis, they occur commonly in childhood [3]. Patients with Amyand’s hernia remain asymptomatic, and during the inguinal hernia repair procedure appendix is found with or without inflammatory changes. The standard surgical treatment for Amyand’s hernia consist both appendectomy and hernia repair [4].

In this case report, we present an 11-month-old male infant in whom Amyand’s hernia was discovered intraoperatively, where the appendix was found densely adhered within the right inguinal hernia sac.

The manuscript is written as per the SCARE guideline [5].

## Case presentation

An 11-month-old male was brought to our surgical center with a history of swelling in the right inguinal region noted since birth. The swelling was described as more prominent during episodes of crying and coughing and spontaneously reduced at rest. There was no associated history of fever, vomiting, abdominal pain, abdominal distension, or urinary symptoms such as dysuria or burning micturition. The child had been feeding well and showed no signs of systemic illness.

On physical examination, a soft, reducible, non-tender mass was palpable in the right inguinal region. The mass increased in size with Valsalva-like maneuvers (crying/coughing) and reduced on relaxation. There were no signs suggestive of strangulation or incarceration, such as skin discoloration, irreducibility, or tenderness. The contralateral inguinal region was normal. The rest of the physical and systemic examinations were within normal limits.

Laboratory evaluation showed no evidence of systemic infection (Table 1). Ultrasound of the inguinal region confirmed a right-sided inguinal hernia, showing bowel loops within the hernial sac, but the appendix was not clearly visualized. No signs of bowel wall thickening, free fluid, or compromised vascularity were noted.

**Table 1:** Blood investigations

Test	Result	Unit	Reference range
Total leukocyte count	13 900	/cmm	6000–17 500
Hb	11.5	gm%	10.5–13.5
Platelets	482 000	/cmm	214 000–459 000
Urea	5.3	mmol/l	2.5–7.1
Creatinine	30	micromole/l	15–37
PT	13.6	seconds	11–13.5
INR	1.04		0.8–1.2
Sodium	138	mEq/l	135–145
Potassium	4.7	mEq/l	3.5–5.1

Based on clinical and radiological findings, a diagnosis of reducible right inguinal hernia was made. Differential diagnoses, including hydrocele, lymphadenopathy, undescended testis, and strangulated or incarcerated hernia, were considered and systematically ruled out based on clinical reducibility, lack of tenderness, absence of systemic signs, and supportive imaging.

**Figure 1:**
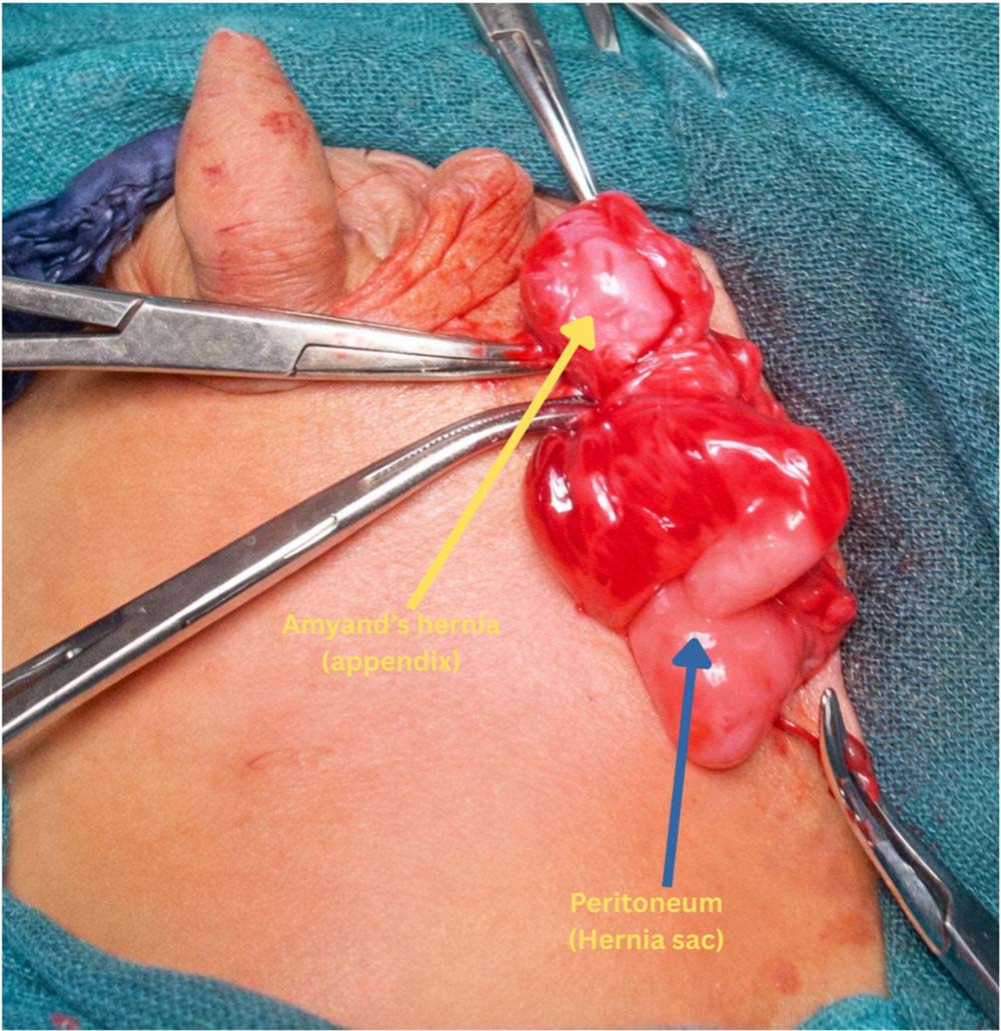
Intraoperative photo showing the densely adhered appendix to the inner wall of hernia sac.

The child was taken up for elective herniotomy. Intraoperatively, the hernial sac was found to contain a portion of the large bowel, including the cecum and a macroscopically normal appendix. However, the appendix was densely adhered to the inner wall of the hernial sac (Fig. 1). In view of the adherent appendix and the risk of future inflammation or complications, an appendectomy was performed in conjunction with the herniotomy. There was no contamination of the surgical field, and standard aseptic technique was maintained throughout.

Postoperatively infant tolerated the procedure well. Postoperative recovery was uneventful with no signs of infection or complications. Oral intake was resumed within 24 hours, and the patient was discharged on the third postoperative day in stable condition. At 4 month follow-up examination on outpatient basis showed complete recovery without recurrence or any complications.

## Discussion

Amyand’s hernia got its name to honor Claudius Amyand, surgeon who first described the presence of a perforated appendix within the inguinal hernia sac of an 11-year old boy and performed a successful trans-herniotomy appendectomy in 1735. It is mostly present on the right side, probably as a consequence of the normal anatomical position of the appendix, and also because right inguinal hernias are common than left-sided hernia [6]. However, there are some exceptions where the appendix is on the left side: situs in-versus, intestinal malrotation, a very loose cecum, or a large appendix [7]. Amyand’s hernia has been reported in individuals aged from 3 weeks to 92 years [6], but it is three times more common in children, primarily because of the presence of a patent processus vaginalis [3].

Though Amyand’s hernia often presents with right lower quadrant pain and a tender, irreducible mass in the inguinal or inguinoscrotal area, which can initially be misidentified as an incarcerated hernia [4],but in our case, there was no history of abdominal pain. The appendix can be present without inflammation, as in our case, or could become inflamed with subsequent perforation and abscess if there is delayed diagnosis [8].

As in our case, most Amyand’s hernias are diagnosed intraoperatively because pre-operative diagnosis is rare [3]. Preoperative diagnosis can be made by sonography, as it is often a cheaper and safer option and provides real-time imaging without the ionizing radiation associated with computed tomography (CT), making it a safer option for use in pediatric and pregnant patients. However, preoperative diagnosis based on ultrasound alone is heavily dependent upon the technical skill of the operator, and as such remains a relatively unreliable modality, so positive findings should be confirmed using CT if necessary [2–4]. Surgery can be both diagnostic and therapeutic when diagnosis remains unclear.

In 2007, Losanoff and Basson created a classification scale to identify and treat Amyand’s hernias [2].

**Table:** 

Classification	Description	Surgical management
Type 1	Normal appendix in an inguinal hernia	Hernia reduction, mesh repair, and appendectomy in young patients
Type 2	Acute appendicitis in an inguinal hernia, without abdominal sepsis	Appendectomy, primary repair of hernia without mesh
Type 3	Acute appendicitis in an inguinal hernia, abdominal wall or peritoneal sepsis	Laparotomy, appendectomy, primary repair without mesh
Type 4	Acute appendicitis in an inguinal hernia, with abdominal pathology	Manage as Type 1–3, investigate and treat second condition as appropriate

In pediatric patients, the management of Type 1 Amyand’s hernia differs from that in adults. Standard treatment consists of herniotomy without mesh placement due to the low recurrence risk in children and concerns regarding foreign body implantation in infants. In our case, although the appendix appeared macroscopically normal, it was densely adherent to the hernia sac, making reduction difficult; therefore, appendectomy was performed along with herniotomy. The decision to remove a non-inflamed appendix in Type 1 Amyand’s hernia remains controversial. The advantages include prevention of recurrent appendicitis within the hernia sac and avoidance of reoperation, whereas disadvantages include conversion of a clean procedure into a clean-contaminated field with potential infectious complications. Histopathological examination of the appendix was not performed in this case.

## Conclusion

Amyand’s hernia is a rare condition where diagnosis remains as per operative findings. Hence, it demands more diligent effort by surgeons. The choice of surgery is surgeon-dependent, based on presentation and difficulties encountered, as well as the therapeutic options available.

## Peer and provenance statement

The article was externally peer-reviewed.
